# Cocaine Poisoning—Magnan’s Symptoms

**Published:** 1897-02

**Authors:** 


### COCAINE POISONING: MAGNAN'S SYMPTOM.

Rybakoff, at a meeting of the Neurological Society of Moscow (*Neurolog. Centralblatt*, August, 1896), insisted on the diagnostic value of the symptom of chronic cocaine poisoning described by Magnan. This is an hallucination of common sensation; the patient complains of feeling some foreign body under the skin. In some cases the foreign bodies felt were like grains of sand, in others slightly larger; generally they were described as more or less rounded, and gave rise to complaints of microbes, worms, crystals, etc., situated just under the skin. While other symptoms of chronic cocaine poisoning occur also in alcoholism and with other poisons, Magnan's symptoms seems to occur only with cocaine. It has, therefore, a real diagnostic value, especially in cases where the patient is unwilling to admit having used cocaine. Where cocaine is extensively used in surgery and dentistry, the appearance of Magnan's symptom is a valuable indication for the immediate cessation of the drug. Korsakoff reported a case in which a woman suffering from multiple neuritis complained of "worms in the skin." On inquiry it was found that vaginal tampons containing cocaine had been freely used. The omission of these was followed by amelioration of the symptom.—*Therapeutic Gazette.*

